# Possible reasons why female physicians publish fewer scientific articles than male physicians – a cross-sectional study

**DOI:** 10.1186/s12909-015-0347-9

**Published:** 2015-04-02

**Authors:** Ann Fridner, Alexandra Norell, Gertrud Åkesson, Marie Gustafsson Sendén, Lise Tevik Løvseth, Karin Schenck-Gustafsson

**Affiliations:** 1 Department of Psychology, Stockholm University, SE-10691 Stockholm, Sweden; 2 Karolinska Institutet, Centre of Gender Medicine, Stockholm, Sweden; 3 Division of Mental Health Care, Department of Research and Development and Faculty of Medicine, St. Olav’s University Hospital, NTNU, Trondheim, Norway

**Keywords:** Physicians, Biomedical research, Publications, Work control, Burnout, Women physicians

## Abstract

**Background:**

The proportion of women in medicine is approaching that of men, but female physicians are still in the minority as regards positions of power. Female physicians are struggling to reach the highest positions in academic medicine. One reason for the disparities between the genders in academic medicine is the fact that female physicians, in comparison to their male colleagues, have a lower rate of scientific publishing, which is an important factor affecting promotion in academic medicine. Clinical physicians work in a stressful environment, and the extent to which they can control their work conditions varies. The aim of this paper was to examine potential impeding and supportive work factors affecting the frequency with which clinical physicians publish scientific papers on academic medicine.

**Methods:**

Cross-sectional multivariate analysis was performed among 198 female and 305 male Swedish MD/PhD graduates. The main outcome variable was the number of published scientific articles.

**Results:**

Male physicians published significantly more articles than female physicians *p* <. 001. In respective multivariate models for female and male physicians, age and academic positions were significantly related to a higher number of published articles, as was collaborating with a former PhD advisor for both female physicians (OR = 2.97; 95% CI 1.22–7.20) and male physicians (OR = 2.10; 95% CI 1.08–4.10). Control at work was significantly associated with a higher number of published articles for male physicians only (OR = 1.50; 95% CI 1.08–2.09). Exhaustion had a significant negative impact on number of published articles among female physicians (OR = 0.29; 95% CI 0.12–0.70) whilst the publishing rate among male physicians was not affected by exhaustion.

**Conclusions:**

Women physicians represent an expanding sector of the physician work force; it is essential that they are represented in future fields of research, and in academic publications. This is necessary from a gender perspective, and to ensure that physicians are among the research staff in biomedical research in the future.

## Background

During the last few decades, the number of female physicians in European countries has increased considerably and we are now approaching an even distribution between female and male physicians [1]. In Europe and North America, since the 1970s, women have entered academic fields of medicine in increasing numbers [2]. Nevertheless, female physicians are still struggling to reach the highest positions in academic medicine [3,4]. Women represent a persistently low proportion of faculty in senior and leadership roles in the field [5-8]. Female physicians face barriers to academic career progression [9] and are under-represented, compared to their male counterparts, at senior levels. For example, in the UK, one in five medical schools do not have a female professor [10], only two out of the 33 heads of medical schools are women, and at a professorial level only 11% of clinical academics are women [11]. Previous research in the field of academic medicine shows that the disparities between the genders in academic medicine are due to the fact that male physicians have a higher publishing rate than their female colleagues [12] i.e. female physicians lack this important qualification for promotion in academic medicine. One reason might be that women obtain fewer grants [13,14] and are often found to be financially disadvantaged when variations in the distribution of work time and productivity are considered [15,16]. Women are also underrepresented on the editorial boards of major medical journals [17]. In the UK, the number of female first authors in prestigious journals has increased but not as female senior authors, and we do not know if these authors have been clinical physicians [18]. The gender publication gap also exists among younger academic hospital physicians who might be expected to emphasize work-life balance and display less gender publication bias [19]. Furthermore, female physicians are less likely than male physicians to have a mentor, and this lack of mentoring could negatively affect women’s careers [20-24]. It has been reported that women with mentors produce more published work, spend more time on research activity, and have higher overall career satisfaction than those without mentors [24]. The most common obstacles identified by women faculty members in achieving career advancement goals have been shown to be clinical workload, insufficient financial support for research, and insufficient institutional support [20,25-27].

Throughout the world women leave their academic careers in far greater numbers than their male colleagues [4,28-30]. One reason may be that women suffer discrimination due to gender. For example, it has been shown that peer reviewers for research grants cannot judge scientific merit without considering gender [28-31]. The peer reviewers overestimate male achievements and/or underestimate female performance, as shown throughout multiple-regression analyses of the relationship between defined parameters of scientific productivity and competence scores, and in a meta-analysis of the peer-review process.

There is a known hierarchy within medical academic research, and this structure precludes female physicians from moving upward in the field of academic medicine [32]. The hierarchical structure in academia takes advantage of female physicians’ lack of confidence in terms of proficiency and academic achievement. Furthermore, recruitment processes are experienced as excluding women [32].

When women seek senior positions or research grants and their former advisors are co-authors on later scientific papers the women might be judged as being dependent on the former supervisor, while men might be seen as new partners [3]. To establish an academic career, younger researchers need to show their independence, and it is difficult to get your own research grants if you have been assessed as dependent [28]. However, we lack research about whether collaboration with a former advisor has any impact on future publications.

In the US, the under-representation of female physicians in higher ranks of academic medicine has been found to be partly related to work time and high stress [20]. Competing demands of research, teaching and clinical work are difficult to cope with for all clinical academics [33]. One of the most common obstacles identified by women faculty members in achieving career advancement goals is clinical workload. In a Polish longitudinal study, low levels of stress and burnout experienced by the physician were prerequisites for success in a medical career [34]. Hence, it is important to consider the influence of exhaustion on the number of publications. Job demands have been found to be the most important antecedents of exhaustion [35-37].

In societies that value gender equality, it is unacceptable for medical research to lag behind in this respect. Although research is supposed to be objective, in reality it is influenced by the researcher [38], which is why it is relevant that the female population is represented by female medical researchers.

### Aim of the study

Discovering which factors interfere with the biomedical research of female physicians is a necessary step for increasing the number of active female physician scientists and for ensuring that physicians are among the research staff of the future. Previous studies have highlighted gender differences in academic medical publications. This is, to our knowledge, the first study that statistically analyses work-related factors related to this publication gap where the number of publications is the outcome variable. We wish to examine gender differences among clinical physicians working at a university hospital and who also have a PhD. Having control over the amount of work assigned, setting one’s own work hours, and continuing to collaborate on research with a former PhD advisor are hypothesized to be associated with a higher rate of publication. In contrast, exhaustion is hypothesized to be associated with a lower incidence of academic publication.

## Methods

### Settings

The HOUPE study is a longitudinal research program concerning work-related health, organizational culture, career paths and working conditions in university hospitals in several European countries. In Sweden, the study was carried out in 2005 at large governmental university hospitals in the central part of the country. A letter was sent to each eligible physician. The letter contained a personal password and log-on information to access the web-based questionnaire. Four reminders to participate were sent by email. In addition, a paper version of the questionnaire was sent in order to provide an alternative for those who were reluctant to respond electronically. The study was reviewed and approved by the Regional Ethics Board (Stockholm) on 8 December 2004, number 04-913/2. Further details about the HOUPE study are presented in work by Fridner et al. cited in the references [39-41].

### Participants

Eligible physicians (permanently employed and actively working) received written information about the survey, together with endorsements by the director of the University Hospital, the chairperson of the local medical association, and the project manager. The response rate was 59.8%, and the response rate for female physicians was slightly higher than for male physicians (65% and 53.7% respectively). The present paper includes the 503 physicians who stated that they had a PhD, 198 (51.4%) of the female physicians, and 305 (66.9%) of the male physicians.

### Dependent variable

The outcome variable, *number of published articles*, was assessed by using the Physician Career Path Questionnaire (PCPQ) [3]. In the present study, a dichotomization was made between < =16 and >16 articles. We chose this cut-off point since having more than 16 published articles demonstrates a continuous record of publishing following a PhD degree, and entitles a person to apply for associate professorship in Sweden [42].

### Independent variables

The independent factor of *Are you still doing research with your former thesis advisor?* (with answers divided into “Yes” or “No”), and *academic position* were both assessed by using PSPQ [3].

*Age* groups were divided into three categories: “younger than 40 years of age”, “40-54 years of age” and “55 years of age or older”. Factors regarding *civil status* (in a relationship, married/living with a partner or single, divorced/separated), *number of children, number of children under the age of 18 years living in the household,* and *number of children before gaining PhD* degree were also included in the analysis.

*Exhaustion* was measured according to a five-item scale (α = 0.80, e.g. “After my work, I usually feel worn out and weary”) based on the “Mini Oldenburg Burnout Inventory” (MOLBI) [36]. This dimension was measured by four answer options: “totally agree”, “agree”, “disagree” and “totally disagree”.

We assessed items from the Nordic Questionnaire for Psychological and Social Factors at Work (QPS-Nordic) [43] concerning *control over the amount of work* assigned and setting one’s own work hours, each measured using a scale with a 5-point range from “never or very seldom” (1) to “very often”(5), with α = 0.83.

### Statistical analysis

The socio-demographics and other characteristics of the physicians with a PhD were assessed by numerical count and percentages, as well as by means and standard deviation. Chi square tests for discrete variables and independent-sample *t*-tests for scales were used to compare female and male physicians who had a PhD degree. First, bivariate logistic regression was performed with each of the potential independent variables to find the significant and near significant unadjusted odds ratios (ORs) with respect to the outcome. We performed multiple logistic regressions (MLOGR) to identify parsimonious sets of non-colinear independent variables that explained the largest amount of variance for the outcome variable. This analysis was conducted separately for the female and male physicians. Statistical analysis was performed using SPSS software (SPSS version 21, SPSS Inc., Chicago, Illinois).

## Results

The characteristics of physicians with a PhD are reported with frequency distributions in Table 1.

**Table 1:** **Comparison of demographic characteristics and perceptions of health and work-related factors of female and male physicians with a PhD**

Characteristics		Female	Male	p ^ † ^
Number of respondents		198	305	
Age group				NS
	<40	12 (6.1%)	15 (4.9%)	
	40-54	123 (62.1%)	165 (54.3%)	
	>54	63 (31.8%)	124 (40.8%)	
	Missing data	0	1	
Civil status				0.003
	In a relationship	157 (82.2%)	272 (91.6%)	
	Single	34 (17.8%)	25 (8.4%)	
	Missing data	7	8	
Number of children				0.013
	0	26 (13.9%)	29 (9.8%)	
	1 or 2	102 (54.5%)	135 (45.5%)	
	>2	59 (31.6%)	133 (44.8%)	
	Missing data	11	8	
Children before PhD				0.033
	Yes	134 (80.7%)	193 (69.7%)	
	No	27 (16.3%)	74 (26.7%)	
	Not relevant	5 (3.0%)	10 (3.6%)	
	Missing data	32	28	
Collaboration with former PhD advisor				NS
	Yes	63 (32.6%)	75 (25.6%)	
	No	119 (61.7%)	208 (71.0)	
	Not relevant	11 (5.7%)	10 (3.4%)	
	Missing data	5	12	
Number of publications				0.001
	0-15	109 (56.5%)	95 (31.6%)	
	>16	84 (43.5%)	206 (68.4%)	
	Missing	4	5	
Work-related factors				
	Control at work, mean (SD)	2.62 (0.98)	3.09 (0.99)	0.001
	Missing data	8	10	
Psychological distress				
	Exhaustion, mean (SD)	2.69 (0.53)	2.45 (0.55)	0.001
	Missing data	6	7	

Values are number and (%) of respondents unless otherwise specified.

^†^p-values measured by χ^*2*^*-*test.

Male physicians had published significantly more scientific articles, χ^2^ (_2_) = 30.113 (p = 0.001). There were no significant differences in age, χ^2^ (_2_) = 4.160 (p >0.05, ns). Significantly more men compared to women were in a relationship χ^2^ (_1_) = 9.630 (p = 0.002). A χ^2^-test also showed a significant relationship between gender and number of children χ^2^ (_2_) = 8.730 (p = 0.013), in that male physicians had more children than their female colleagues, and the same was true for the number of children under 18 years currently in the household χ^2^ (_2_) = 8.691 (p = 0.013). The number of physicians who already had children before taking a PhD was also examined in the study, and the result showed a significant difference χ^2^ (_2_) = 6.797 (p = 0.033). More women compared to men had their children before taking a PhD. Male physicians held academic positions to a significantly higher degree than female physicians χ^2^ (3) = 10.017 (p = 0.018).

There were no significant differences as regards collaboration with a former thesis advisor, χ^2^ (_2_) = 4.948 (p = 0.05, ns).

In the study, sex differences relating to control over one’s work were analysed by comparing, for each gender, the self-estimated level of control over the amount of work assigned, work pace, the possibilities for breaks, and the extent to which it was possible to set one’s own work hours. The results are presented in Table 1. Male physicians reported greater freedom to control their work in terms of work-time flexibility and work pace, t (483) = 5.138 (p = 0.001). Furthermore, a comparison of the level of exhaustion in women and men was made, which found a significant difference between female and male physicians, t (480) = −4.748 (p = 0.001), in that female physicians experienced greater exhaustion.

On bivariate logistic regression, for both genders, the non-adjusted risk estimates for various factors suggested a significant relationship between the quantity of scientific work published and age, academic position, collaboration with a former PhD thesis advisor, control over work pace, and number of children living in the household under 18 years old (Table 2). Exhaustion had a significant association with the number of scientific publications for female physicians only.

**Table 2:** **Bivariate unadjusted logistic regression with number of publications as the outcome variable**

	Female			Male		
	OR	95% CI	p	OR	95% CI	p
Age	5.51	2.76-10.99	<.000	2.95	1.87-4.64	<.000
Children under 18 living in the household	0.44	0.29-0.68	<.001	0.71	0.57-0.88	.002
Academic position	2.86	1.97-4.15	<.001	2.68	2.10-3.44	<.001
Collaboration with former PhD advisor	3.47	1.74-6.91	<.001	2.76	1.60-4.76	<.001
Control over work pace	1.61	1.12-2.3	.010	1.81	1.39-2.34	<.001
Exhaustion	0.31	0.15-0.63	0.001	0.80	0.52-1.24	NS

In Table 3 an adjusted Odds Ratio (OR) and Confidence Intervals (CI) from the multiple logistic regression models are presented, which compare research publications among female and male physicians with a PhD. Age and academic position were significantly related to a higher rate of published articles for both female and male physicians (OR = 3.22; 95% CI 1.36-7.61 and OR = 2.30; 95% CI 1.31-4.04) and (OR = 3.09; CI 1.87-5.12 and OR = 2.54; CI 1.92-3.36) respectively. Female and male physicians collaborating with former PhD advisors published more articles than those not collaborating with former advisors: female physicians (OR = 2.97; 95% CI 1.22–7.20) and male physicians (OR = 2.10; 95% CI 1.08–4.10). Control at work was significantly associated with a higher number of published articles for male physicians (OR = 1.50; 95% CI 1.08–2.09) but not for females. Exhaustion had a significant negative association with the number of published articles among female physicians (OR = 0.29; 95% CI 0.12–0.70), whilst the publishing rate among male physicians was not significantly affected by exhaustion.

**Table 3:** **Multiple logistic regression models among female and male MDs/PhDs with number of published research articles as the outcome variable**

Group	Predictors	OR	95% CI	p
***Female***				
Demographic factors	Age	3.22	1.36-7.61	0.008
Work-related factors	Academic position	3.09	1.87-5.12	<.001
	Collaboration with former PhD advisor	2.97	1.22-7.20	0.016
	Control at work	1.02	0.63-1.65	NS
Psychological distress	Exhaustion	0.29	0.12-0.70	0.006
***Male***				
Demographic factors	Age	2.30	1.31-4.04	0.004
Work-related factors	Academic position	2.54	1.92-3.36	<.001
	Collaboration with former PhD advisor	2.10	1.08-4.10	0.030
	Control at work	1.50	1.08-2.09	0.017
Psychological distress	Exhaustion	1.13	0.64-1.97	NS

Multiple logistic regression with adjustment for non-significant socio-demographic factors: civil status and number of children.

CI = Confidence Interval. NS = Statistically non-significant (p >0,05).

## Discussion

This study investigated factors that impede or support scientific publication among female and male physicians with a PhD working at a university hospital. Our findings show that male physicians published significantly more scientific articles compared to female physicians. Having control over one’s work, still collaborating with former thesis advisors, and not experiencing exhaustion were all associated with a higher number of published articles.

To our knowledge, this is the first report to compare work conditions and academic production among female and male physicians with a PhD (in Sweden the granting of a PhD in academic medicine requires the candidate to have a minimum of six peer-reviewed published articles) working in the same university hospital. The advantages of quite a high response rate and a large sample size strengthen the power of this study. Women publish less, and one reason might be an uneven distribution of research funds between women and men, in that women are given less funding than their male colleagues. In reviewing applications to the Swedish Council for Working Life and Social Research (FAS), the analysis showed that female applicants were less likely than men to get research grants. In turn, men’s applications received higher ratings than women’s in peer reviews [44]. However, women were less likely to apply for funding than men and they request smaller amounts of money [45].

There have been suggestions that female researchers’ careers are delayed due to childbearing after being awarded the PhD, when one is supposed to start one’s career. The biological clock clashes with the tenure clock. However, in our population, 80.7% of the female physicians had children before receiving their PhD degree; i.e. they had already simultaneously engaged in both work as physicians and the writing of articles for their thesis. Previous research by Fridner [3] confirms this by showing that in fact the male physicians were more likely to become parents after taking a PhD than their female colleagues. We always control for age, civil status and amount of children when we use binary logistic regression. Most of the literature on women, academic productivity and children are from the 90s. In Sweden fathers, especially well educated, are on parental-leave and stay at home from work when their children are sick. Therefore, we did not find it necessary to include child-care in the hypothesis, and our results show that children did not turn out to be a significant factor for non-publication.

It was hypothesized that collaboration with former PhD advisors would be associated with the publication of a greater number of articles. Through such collaboration, physicians had access to a role model. A high quality relationship with one’s advisor may also alleviate the influence of job demands on job strain [37]. We found that both female and male physicians published more when they had continued to collaborate with a former thesis advisor, compared to those who did not. This result is appealing, considering that previous research indicates that male advisors tend to favour male PhD students with the resources and tools they need for advancement in their academic careers. Women have more often reported that their mentor has taken credit for their work [29].

The results in this study showed that physicians with more control over work time and work pace had a higher publishing rate. It was shown that men, to a greater extent than women, experienced this control. Such control gave them an opportunity to plan their time when conducting clinical research and writing scientific articles. This can be related to earlier research indicating that the work environment influences one’s creativity and innovation [46]. Hence, it can be presumed that positive work conditions promote achievements in research. Since the male physicians in the present study felt more satisfied regarding control at work, it is possible that this is linked to the higher publishing rate among men. For female physicians, no such relation between publication and control over their work was found. Furthermore, it can be assumed that the feeling of control also diminishes the risk of exhaustion, at least for male physicians. This is the first time that a study in this field has shown that lack of control over work time and work pace may have a negative impact on publishing rate for physicians in academic medicine. This result is in line with earlier research which found the work environment to be strenuous, especially for women, due to hierarchical structures in academic medicine [32]. Previous research has revealed that there is a will in academic medicine to move towards non-hierarchical structures. Therefore, organizations with a flatter structure could promote more innovative and creative work environments [45]. Other research recommends an institutional approach that acknowledges the impact of hidden gender-linked biases in academic medicine that marginalize women and negatively affect their well-being [47].

It is known that women physicians experience greater exhaustion than men [48-51], and this is confirmed in our study. To our knowledge, no earlier research has found that exhaustion may prevent women from publishing scientific articles. In the present study it was hypothesized that exhaustion could negatively affect a person’s publishing rate. It seemed unexpectedly the direction could be that low publication rate would increase the risk of exhaustion. The study found that female physicians suffered from exhaustion to a greater extent than their male colleagues. In addition, exhaustion was a risk factor for women’s lower publishing rate. Previous research has shown that both interpersonal and social factors predict exhaustion among physicians [52]. It is important for hospital organizations to prevent these kinds of problems. Today, young women represent a larger proportion of the total number of medical students than previously, and thus more women are likely to become future researchers in biomedical medicine. Clinical research tends to become deficient when female physicians are not included in networks of academic medicine. Moreover, they do not achieve the same publishing rate as male physicians and they are not promoted to high positions [53,54]. As female physicians are more exposed to exhaustion, this factor may also have a negative impact on future clinical research.

### Limitations

Our data is cross-sectional, and thus excludes conclusions about the directionality of the associations we have found. The results are from one hospital, but this academic hospital demands a PhD from physicians in order to qualify for a major position. In the future, this might be the case for all university hospitals, which will increase competition within academic medicine. Due to the anonymity of the data, a multilevel analysis, in which departments and units could be separated, has not been possible.

## Conclusions

Considering that women physicians are an increasing proportion of the physician work force it is essential that they are also represented in the future field of research and academic publications. Control over work, and issues of work-related health such as exhaustion, need to be acknowledged within academia in order to achieve this aim.
